# A multimodal logistics service network design with time windows and environmental concerns

**DOI:** 10.1371/journal.pone.0185001

**Published:** 2017-09-21

**Authors:** Dezhi Zhang, Runzhong He, Shuangyan Li, Zhongwei Wang

**Affiliations:** 1 School of Traffic & Transportation Engineering, Central South University, Changsha, Hunan, China; 2 School of Transportation and Logistics, Central South University of Forestry and Technology, Changsha, Hunan, China; Beihang University, CHINA

## Abstract

The design of a multimodal logistics service network with customer service time windows and environmental costs is an important and challenging issue. Accordingly, this work established a model to minimize the total cost of multimodal logistics service network design with time windows and environmental concerns. The proposed model incorporates CO_2_ emission costs to determine the optimal transportation mode combinations and investment selections for transfer nodes, which consider transport cost, transport time, carbon emission, and logistics service time window constraints. Furthermore, genetic and heuristic algorithms are proposed to set up the abovementioned optimal model. A numerical example is provided to validate the model and the abovementioned two algorithms. Then, comparisons of the performance of the two algorithms are provided. Finally, this work investigates the effects of the logistics service time windows and CO_2_ emission taxes on the optimal solution. Several important management insights are obtained.

## Introduction

The growing specialization and internalization of the world trade have led to increasing distances between suppliers, producers, and final customers. This development results in the increasing volumes of global transportation operations during the last decade [1,2]. Logistics service providers or transport operators should apply a combined transportation method to potentially maximize the corresponding transportation service.

The freight transportation services of multimodal transportation involve various transportation modes, such as road, rail, maritime, air, and pipeline. The number of transportation alternatives can be increased by using different transportation modes and by combining them in multimodal transportation chains. Multimodal transportation not only promotes advantages of each transportation mode but also releases their disadvantages.

The increasing volume of freight transportation leads to regional economic development; however, this volume also reveals certain negative effects [3]. Freight transportation is widely considered a major contributor to climate change, and global warming is attributed to various pollution emissions. Freight transportation contributes to approximately 5.5% of global greenhouse gas emissions [4]. CO_2_ emissions from transportation amount to 93% of the total pollution emissions during logistics service activities, whereas warehousing covers only 7% [5,6]. Therefore, creating an environmentally sustainable logistics system is important and urgent. Green logistics focuses on improving logistics service efficiency, decreasing logistics cost, and reducing environmental externalities (e.g., CO_2_) to achieve a sustainable balance between economic, environmental, and social objectives [3,7,8]. Logistics network design and vehicle routing are two important methods among the green logistics initiative measures for decreasing CO_2_ emissions [3,5,9]. Aronsson and Brodin demonstrated that logistics efficiency and cost are related to not only the structure of supply chains but also the logistics network design and logistics infrastructure [10]. Logistics service network design, as an important component of a global supply chain logistics system, is a strategic issue involving logistics facility planning and combined transportation mode planning. Therefore, designing a multimodal logistics service network with customer service time windows and environmental costs is an important and challenging issue.

Numerous related studies focused on logistics network design and can be classified into the following three aspects in terms of modeling methodology and contexts: supply chain, regional, and green logistics network design.

In terms of the supply chain logistics network design, the related research is summarized as follows:

Elhedhli et al. [11] presented a large-scale network design model of an automotive company, which considered the outbound supply chain design that considered the choices in transport mode, the location of distribution facilities, and lead time collectively; the researchers subsequently proposed a Lagrangian heuristic algorithm. Huang et al. [12] developed a mathematical model for the multistage optimization of the supply chain of biofuels, which aims to minimize the cost of the entire supply chain of biofuels considering the technology, resource, and demand constraints. Wang et al. [13] proposed a multi-objective optimization model for the green supply chain network design, which sets the environmental investment decisions in the design phase. A multi-objective stochastic optimization model suggested by Jindal and Sangwan [14] attempted to minimize environmental impacts and traditional costs. Santoso et al. [15] investigated the large-scale problem of supply chain network design by stochastic programming method. The green supply chain design model developed by Elhedhli and Merrick [16] incorporated the cost of carbon emissions, aiming to minimize the environmental cost of CO_2_ emission and logistics.

Compared with the supply chain network design, regional logistics is mainly optimized to configure different logistics nodes to improve logistics efficiency and decrease the total social logistics cost. Kim and Barnhart [17] investigated the large-scale problem for service design and presented a model with a time window. Asgari et al. [18] studied the cooperation and competition in the maritime industry and proposed a game-theoretic network design model that considered three scenarios. A hub location–allocation mathematical model in the intermodal logistics network developed by Ishfaq and Sox [19] considered the dynamics of different transport modes through the varied costs during the entire trip. Chang [20] identified the international intermodal routing problem as a multi-objective, multimodal, and multi-commodity flow problem, which contains three important characteristics, namely, transportation economies of scale, multiple objectives and demanded delivery times, and scheduled transportation modes. The mathematical programming model presented by Tyan et al. [21] evaluated the freight consolidation policies in global third party logistics. Wang et al. [22] investigated an optimization model for a two-echelon logistics distribution network design, which is solved by a hybrid algorithm embedded with particle swarm optimization and genetic algorithm (GA); the researchers found that the proposed approach can be readily implemented to assist the logistics operators in reducing operational costs and improving customer service through a real-world case study. Moreover, Wang et al. [23] addressed the hierarchical structure of logistics network optimization problem using a fuzzy-based customer clustering approach; the researchers used a case study in Anshun, China for evaluating the effectiveness of the proposed approach. Wang et al. [24] recently proposed a new linear optimization model to address the collaborative two-echelon logistics joint distribution network. Their findings showed that their proposed model can be organized through a negotiation process via logistics service providers or participants, which can effectively reduce logistics costs and improve the efficiency of the urban logistics system.

Furthermore, Haghani et al. [25] addressed a large-scale multi-modal multi-commodity network problem with a time window for disaster relief operations. Mohammadi et al. [26] investigated a hub location problem from the multimodal hazardous materials and proposed a mixed integer programming formulation. Jansen et al. [27] described an operational planning system, namely, Planungund Optimierung Program. Bock [28] addressed integrated multiple transshipments, and the multimodal transportation was used for a freight transportation network. Alumur et al. [29] explored a multimodal hub location and a hub network design problem considering the multicriteria feature of hub location problems. Moccia et al. [30] addressed the multimodal transportation problem with several features and developed a column generation algorithm.

The traditional logistics network design model mainly focuses on total cost or operator efficiency and only slightly considers external environmental costs. By contrast, the green logistics network design model concentrates on improving logistics service efficiency, decreasing corresponding logistics costs, and reducing externalities while achieving a sustainable balance among economic, environmental, and social objectives [10,11,13,31].

Relevant research on green network design problem has received considerable attention from researchers in recent years. Harris et al. [32] assessed the effect of the traditional cost optimization approach to strategic modeling on overall logistics costs and CO_2_ emissions by considering the supply chain structure and different freight vehicle utilization ratios. Yang et al. [33] presented a bilinear mixed integer programming model, which considers the network conformation, low carbon resource deployment, and cost from carbon tax. Furthermore, Wang et al. [34] analyzed the ecoefficiency of the logistics network integration models in the closed-loop supply chain below low carbon restriction, which aims to minimize waste generation, energy consumption, and economic costs in the location selection and flow configuration using a multi-objective mixed linear program. He and Luo [35] formulated the logistics network design problem using queuing theory and interval number under low-carbon constraints and uncertainty conditions, aiming to minimize carbon emissions and total costs. Shaw et al. [36] studied a green supply chain network design model presented by chance-constrained programming given the uncertainties of the capacities of suppliers, factories, and storehouses and the demands of customers.

To the best of our knowledge, existing related studies integrating the time window requirements of customers and carbon emission costs for multimodal logistics network design remain scarce. The present work aims to fill this gap by focusing on the multimodal logistics service network design considering transport time, transfer time, carbon emission, and time window demanded by customers.

The current work addressed an optimal multimodal logistics service network designed to minimize the system total costs of transportation, transfer, and carbon emission. The following two issues require solutions: (1) determining optimal transportation model between any two adjoining cities and (2) optimal transfer cities investing to improve the corresponding transfer capacities in the entire logistics service networks.

The main contributions of this paper are as follows. First, the current work proposes an optimal model that incorporates CO_2_ emission costs to determine the optimal transfer nodes and transportation modes, which considers transport time, transfer time, carbon emission, and logistics service time window constraints. Second, GA and heuristic algorithm are proposed to solve the above optimal model. Finally, the effects of the logistics service time windows and CO_2_ emission taxes on the optimal solution are also investigated. Subsequently, several important management insights are obtained.

The remainder of this paper is organized as follows. Section 2 describes the constituents of the model. Moreover, Section 3 introduces the model formulation and analyzes the solution algorithm. Section 4 provides a numerical example to exemplify the applications of the model, followed by analysis and discussions. Finally, Section 5 presents the conclusions and recommendations for further studies.

## Problem description

**Fig 1:**
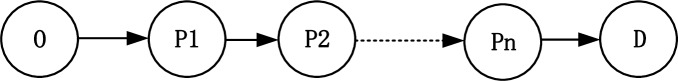
Original transportation map.

In Fig 1, a logistics company sends a shipment of goods from origin place (O) to destination place (D) via intermediary cities during the entire trip. A cost- and time-efficient logistics service network is urgent for each logistics service carrier to satisfy the transport tasks over a large area in the time window demanded by customers with minimum costs.

**Fig 2:**
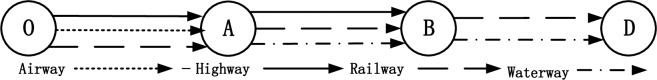
Multimodal logistics service routes.

Owing to the rapid development of transport technology and the standardization of transportation, the multimodal transportation can be used in the logistics service network design. In Fig 2, several alternative transport modes (e.g., highway, railway, and waterway) are available between any two adjoining cities called a city pair. The transport time, transport capacity, transport cost, and carbon emission differ with the transport mode selected for the same city pair. The total transport time of arrival at the destination city cannot exceed the predetermined logistics service time window (the earliest and latest times) by customers. A mode transfer may exist for every transfer city node in the service network, and different transfer times and costs are incurred by the transfer between different transport modes. Moreover, the transfer activities should meet the corresponding transfer feasibility for two transport tools. Thus, selecting the optimal transfer nodes to invest in is necessary to improve the capacity of logistics service network. We aim to select the optimal transport mode combinations and invest in transfer nodes to obtain minimal total costs to deliver goods from their origins to their destinations under the permitting time and capacity constraints.

## Model formulation

### Assumptions

The following basic assumptions are adopted in this work to facilitate the presentation of essential ideas without losing generality:

A1. Only one mode is selected from the alternative transport modes between two adjacent nodes.

A2. The transfer among different transport modes only occur in each transfer node among the logistics service network. Moreover, only one transfer is allowed in each transfer node.

A3. The same transport mode among different nodes demonstrates the same speed.

A4. The transport costs are linear in distance and size of shipment.

### Notations

**Table:** 

**Sets**	
*A*	set of links in the network
*N*	set of nodes in the network
*M*	set of transport modes between the two adjacent cities
*N*' = {*N*/*D*}	set of nodes, except the destination node in the network
*T* = {N/*O*,*D*}	set of transfer nodes in the network
**Parameters**	
[*t*_1_,*t*_*2*_]	permitting logistics service time window; *t*_1_ and *t*_2_ represent the earliest and latest times of arrival at the destination city, respectively
$c_{i,i+1}^{m}$	transport cost from city *i* to *i*+1 by transport mode *m*
$f_{i}^{m,l}$	transfer cost from modes *m* to *l* at city *i*
$e_{i,i+1}^{m}$	unit CO_2_ emission from city *i* to *i*+1 by transport mode *m*
$e_{i}^{m,l}$	unit CO_2_ emission of transfer from transport mode *m* to *l* at the transfer node i
$u_{i,i+1}^{m}$	the capacity of transport mode *m* from city *i* to *i*+1
*v*^*m*^	average speed by transport mode *m*
$t_{i,i+1}^{m}$	transport time from city *i* to *i*+1 by mode *m*
$t_{i}^{m,l}$	transfer time from modes *m* to *l* at city *i*
*K*_*i*_	the fixed investment cost of transfer node at city *i*
Q	shipment of goods from cities *i* to *i*+1
*w*	actual total logistics service time from O to D
*M*	an infinitely large penalty factor
$d_{i,i+1}^{m}$	transport distance from cities *i* to *i*+1 by mode *m*
*E*	total CO_2_ emissions during the entire logistics service chain
*λ*	emission taxes per unit CO_2_ emission
$\mu_{i}^{ml}$	indication variable of transfer feasibility after investment of transfer nodes. The value is 1 if the transfer conditions are permitted at city I; otherwise, 0
${\bar{\mu }}_{i}^{ml}$	the original value of the transfer feasibility before investing a transfer node to improve its transfer capacity
**Decision variables**	
$x_{i,i+1}^{m}$	0–1 variable equals 1 if goods are shipped by mode *m* from cities *i* to *i*+1; otherwise, to 0
$y_{i}^{m,l}$	0–1 variable equals 1 if goods are transferred from modes *m* to *l* at city *i*; otherwise, to 0
*z*_*i*_	0–1 variable equals 1 if the investment is for transfer capacity over city node *i*; otherwise, equals 0

The optimal model of the multimodal logistics service network can be described as follows:

$$\begin{array}{l} \min Z=\sum_{i\in N/D}\sum_{m\in M}c_{i,i+1}^{m}d_{i,i+1}^{m}x_{i,i+1}^{m}Q+\sum_{i\in T}\sum_{m\in M}\sum_{l\in M}f_{i}^{m,l}y_{i}^{m,l}Q+\sum_{i\in T}K_{i}Z_{i}+\\ \lambda (\sum_{i\in N/D}\sum_{m\in M}e_{i,i+1}^{m}d_{i,i+1}^{m}x_{i,i+1}^{m}Q+\sum_{i\in T}\sum_{m\in M}\sum_{l\in M}e_{i}^{m,l}y_{i}^{m,l}Q) \end{array},$$ (1)

subject to

$$t_{i,i+1}^{m}=d_{i,i+1}^{m}/v^{m},\forall i\in T,m\in M,$$ (2)

$$\sum_{i\in N/D}\sum_{m\in M}t_{i,i+1}^{m}x_{i,i+1}^{m}+\sum_{i\in T}\sum_{l\in M}\sum_{m\in M}y_{i}^{m,l}t_{i}^{m,l}=w,$$ (3)

$$\sum_{m\in M}x_{i,i+1}^{m}=1\;\forall i\in T,$$ (4)

$$\sum_{m\in M}\sum_{l\in M}y_{i}^{ml}=1\;\forall i\in T,$$ (5)

$$x_{i-1,i}^{m}+x_{i,i+1}^{l}\geq 2y_{i}^{m,l}\;(\forall i\in T),$$ (6)

$$x_{i,i+1}^{m}Q\leq u_{i,i+1}^{m},\forall i\in T,m\in M,$$ (7)

$$t_{1}\leq w\leq t_{2},$$ (8)

$$y_{i}^{m,l}\leq \mu_{i}^{m,l},\forall i\in T,m,l\in M,$$ (9)

$$\mu_{i}^{m,l}=\left\{ \begin{array}{l} 1\;if\;Z_{i}=1,\forall i\in T,m,l\in M\\ {\bar{\mu }}_{i}^{m,l}\;if\;Z_{i}=0,\forall i\in T,m,l\in M \end{array}\right.$$ (10)

$$x_{i,i+1}^{m}\in \{0,1\}\;(\forall i\in N,m\in M),$$ (11)

$$y_{i}^{ml}\in \{0,1\}\;(\forall i\in T,m\in M,l\in M).$$ (12)

The objective function (Eq (1)) minimizes the total costs, which include five parts, namely, the cost of carbon emissions, transport costs, transfer costs, fixed costs of transfer nodes, and penalty cost of transfer).

Constraint (2) indicates the corresponding transport time between two adjacent cities. Constraint (3) implies the total time equals the transport time plus the transfer time. Constraint (4) specifies that only one mode is selected to transport goods between two adjacent cities. Constraint (5) indicates that the transfer can only occur in each transfer city. Constraint (6) ensures the feasibility of transit transport via transfer node *i*. Constraint (7) ensures that the capacity of transport model m between city *i* and *i*+1 does not exceed. Constraint (8) states that the total logistics service time during the entire trip should meet the requirement of logistics service time windows. Constraint (9) states that the transfer transport should meet the corresponding feasibility at each transfer node. Constraint (10) implies that the value of transfer indication variable is 1; otherwise, the value of the transfer indication variable equals its original value. Constraints (11) and (12) define the decision variables as binary.

According to [31,37,38], the total CO_2_ emission of a logistics service path includes two parts, i.e., the CO_2_ emissions of haul transportation and transfer nodes. The CO_2_ emission of haul transportation is related with transport mode, load, and distance. The total CO_2_ emission (*E*^1^) of transport mode *m* carrying a load of Q (in tons) at a distance of *d* (in km) is calculated as expressed follows:

$$E^{1}=e^{m}*d*Q$$ (13)

where *e*^*m*^ is the average CO_2_ emission factor of transport mode *m* (g/ton-km). However, the CO_2_ emissions of transfer nodes are generally finished by a truck at the transfer yard, thereby maintaining minimal changes among different types of transfer (e.g., railway–road, railway–air, and railway–waterway). The transfer CO_2_ emission (*E*^2^) incurred at certain transfer nodes can be calculated as follows.

$$E^{2}=e_{}^{m,l}*Q$$ (14)

where *e*^*m*,*l*^ is the unit CO_2_ emission factor transfer from transport mode *m* to *l*, and *Q* is the total shipment of transfer. We can use a parameter of CO_2_ emission taxes to convert CO_2_ emissions into monetary units.

### Solution algorithm

#### Algorithm1–GA

John Holland introduced a GA, which is a stochastic algorithm categorized in the class of general purpose search methods that simulate natural elution system processes [39–41]. GAs combine directed and stochastic search methods and can achieve a favorable balance between exploration and exploitation of the search space. The advantages of using GA techniques in solving numerous optimization problems are their robustness, search flexibility, and evolutionary nature [42]. Therefore, the present work addresses the abovementioned optimal model through the GA. The GA process is described as follows:

**Step 1**. **Initialization**

(1) Set the GA parameters: population size (*Pop*), crossover probability (*Pc*), and mutation probability (*Pm*).

(2) Set a stopping criterion, i.e., a predefined number of generations (*Maxgen*).

(3) Set iteration counter n = 0, and create the initial population *pop*_*n*_ = $\{R_{k}^{n}|k=1,2,3,\cdots ,p\}$, in which *p* is the population size, and individual $R_{k}^{n}$ is the *k*th decision scheme in the *n*th generation, i.e., $R_{k}^{n}=\{X_{k}^{n},Y_{k}^{n})$ is the decision vector, with $X_{k}^{n}$ representing the selected transfer city nodes to invest in and $Y_{k}^{n}$ representing the different transport modes, such as railway, highway, and waterway, between two adjacent nodes.

**Step 2**. **Calculation of the fitness value**

Compute the total cost of the multimodal logistics service network value ${\left(Z\right)}_{k}^{n}$ on the basis of Eq (14). Let $fit_{k}^{n}=1/{\left(Z\right)}_{k}^{n}$. For each $R_{k}^{n}$ of *pop*_*n*_, calculate the fitness value $fit_{k}^{n}$ on the basis of ${\left(Z\right)}_{k}^{n}$.

**Step 3**. **Sorting**

Sort the individual $R_{k}^{n}$ of the population *pop*_*n*_ in descending order according to the value fitness $fit_{k}^{n}$.

**Step 4**. **Selection process**

The selection process involves spinning the roulette wheel *pop_size* times each time a single chromosome is selected for a new population, as follows:

(1) Calculate the evaluation function.

We can adopt the rank-based evaluation function as follows:

*Eval*(*Vi*) = *a*(1−*a*)*i*−1, ∀*i* = 1,2,3,⋯,*pop*_*size*.*a* = 0.05 *i* = 1 denotes the best individual, whereas *i* = *pop*_*size* refers to the worst individual, and $\sum_{i=1}^{pop\_size}Eval(V_{i})=1$.

(2) Calculate the cumulative probability *Cum*(*i*) for each chromosome *V*_*i*_, i.e., *Cum*(0) = 0, $Cum(i)=\sum_{j=1}^{i}Eval(V_{j}),$ ∀*i* = 1,2,3,⋯,*pop*_*size*.

(3) Generate a random real number *r* in (0,*Cum*[*pop*_*size*]).

(4) Select the *i*th chromosome *V*_*i*_(1 ≤ *i* ≤ *pop*_*size*); thus, *Cum*(*i*−1) < r ≤ *Cum*(*i*).

(5) Repeat Steps (2)–(4) *pop_size* times to obtain the *pop_size* copies of chromosomes.

In this process, the best chromosomes are duplicated several times, the average chromosomes are retained, and the worst chromosomes are terminated.

**Step 5**. **Implementation of crossover and mutation**

Implement crossover and mutation operations to obtain a new population *pop*_*n*_, and set *n* = *n* + 1. Detailed descriptions of the crossover and mutation operations are presented in Sections 3.3.4.and 3.3.5.

**Step 6**. **Implementation of a termination check for the loop operation**

If (*n*<*Maxgen*), then stop the calculation; otherwise, proceed to Step 2.

**Step 7**. **Production of the final optimal solution**

**Fig 3:**
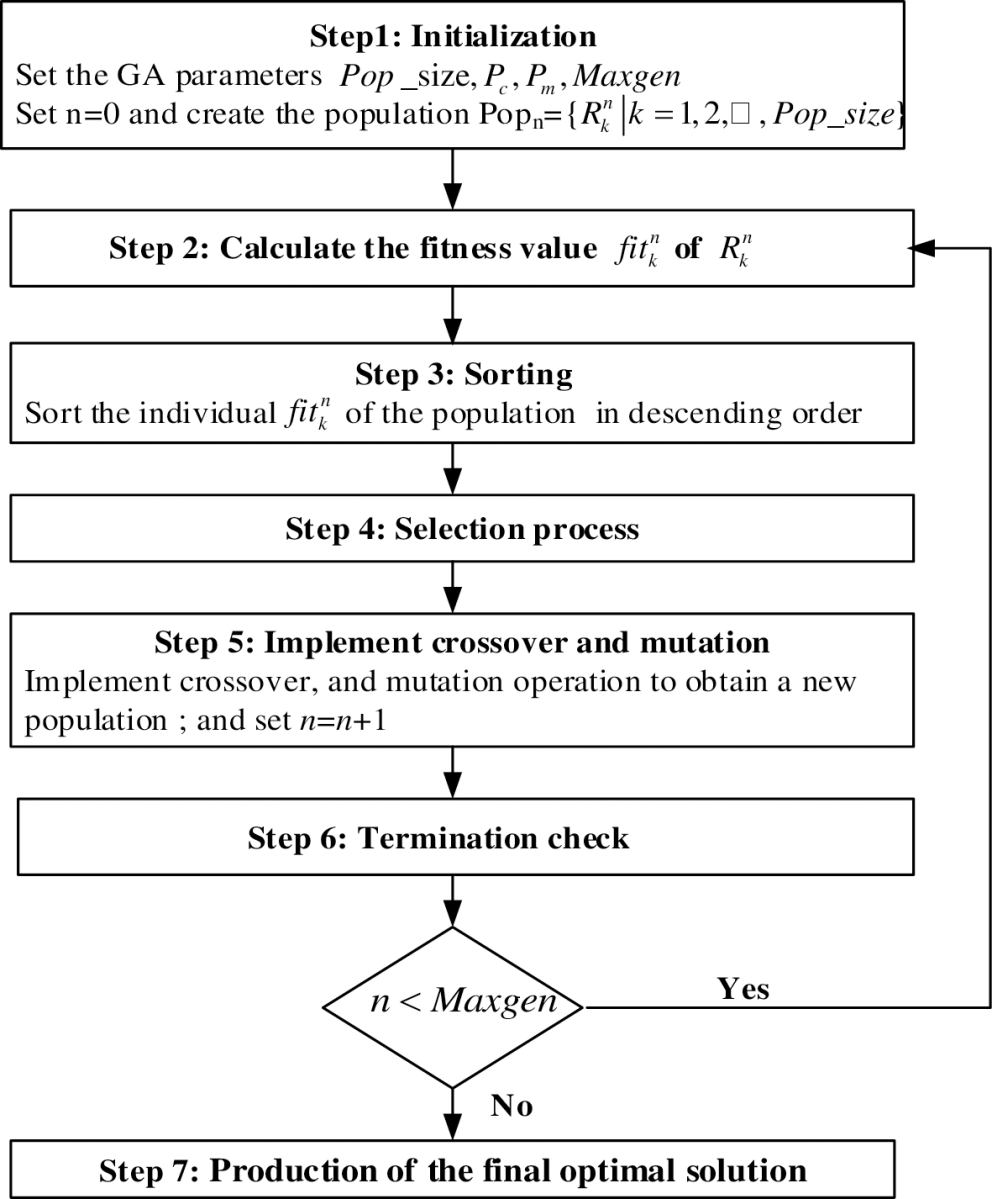
Flowchart of GA.

The flowchart of the above algorithm is depicted in Fig 3.

#### Key element design of GA

The performance of a hybrid GA algorithm is largely influenced by the design of genetic operators and the value of the parameters. In this section, we explain the following three key elements of the GA used in this work: coding, crossover, and mutation calculations.

#### Coding method

**Fig 4:**
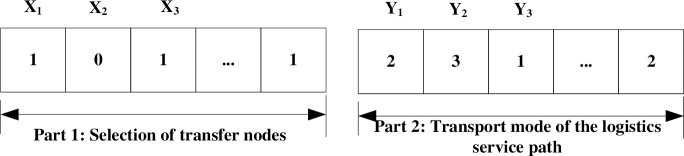
Chromosome of the GA.

The individual chromosome in the GA is composed of two parts (Fig 4).

Part 1 represents the selection of the candidate transfer nodes to improve the transfer capacity, i.e., a value of 1 equates to the condition in which the candidate transfer node is selected to invest; otherwise, the value is 0. In Part 2, the values of 1, 2, 3, and 4 represent the air, railway, highway, and waterway over the logistics service path, respectively.

#### Crossover method

First, we generate a random real number *r* in [0, 1]. Second, we select the given chromosome for a crossover operation if *r* ≤ *P*_*c*_. We repeat this operation *pop_size* times and produce *P*_*c*_∙*Pop*_*size* parents on average.

**Fig 5:**
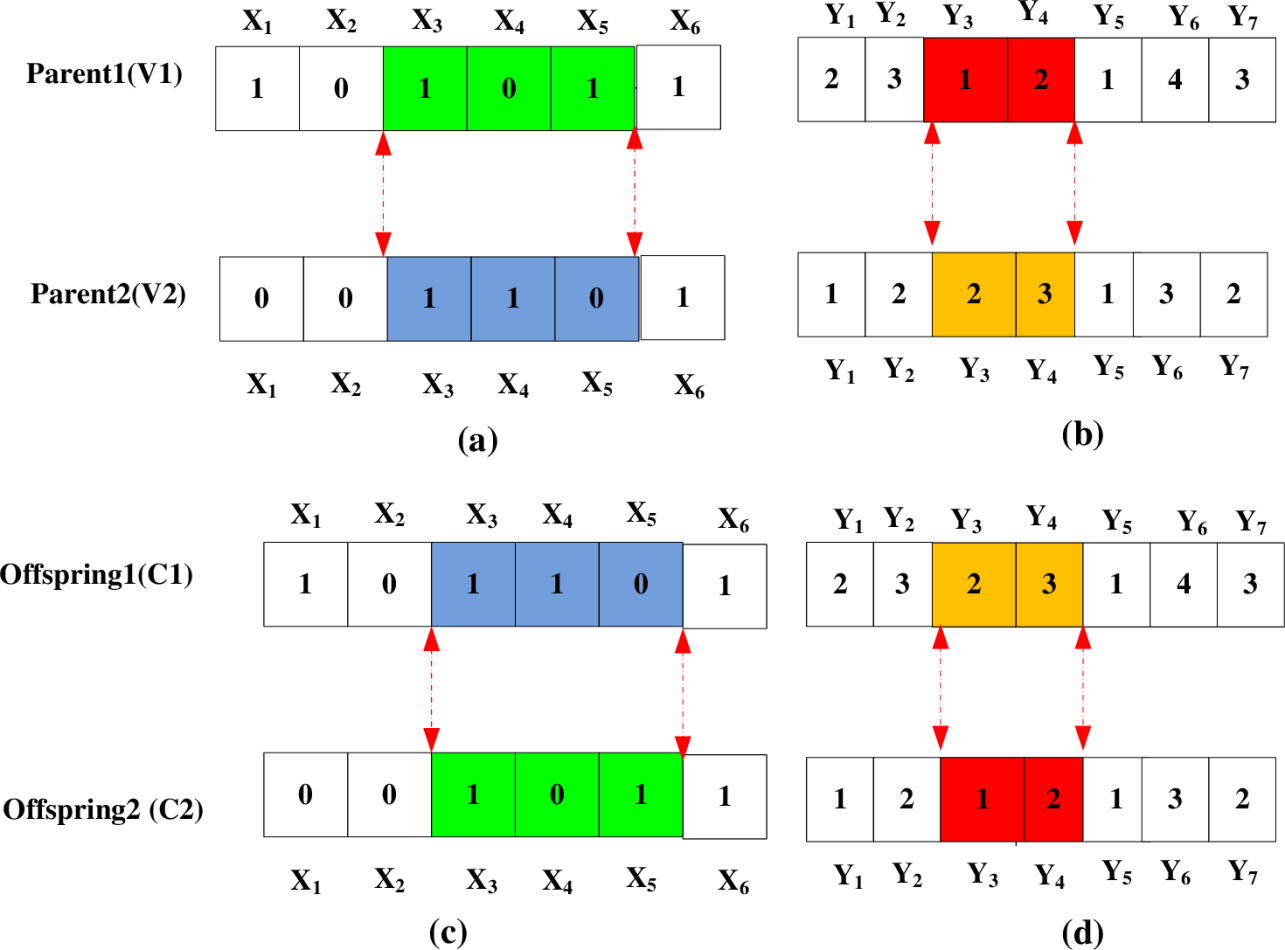
Representation of the crossover operation.

In the present work, we implement a crossover operation for the two parts of the chromosome. The two-point crossover method is adopted for Parts 1 and 2. We provide a simple example consisting of six candidate transfer city nodes and seven city pairs over the entire logistics service path (Fig 5).

#### Mutation method

We perform the mutation on the set of offsprings produced by the crossover operations. We select the given chromosome for mutation if *r* < *P*_*m*_ by generating a random real number *r* in [0, 1]. Let a parent *V*, denoted as a vector **V** = (**X**,**Y**), for mutation be selected.

**Fig 6:**
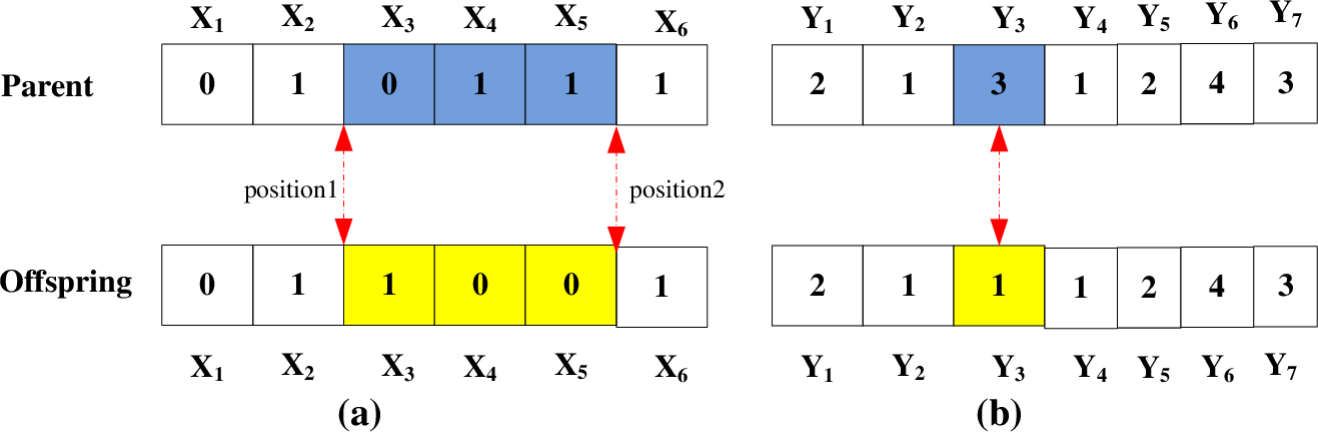
Representation of the mutation operation.

For the first part (i.e., vector X), the two-point mutation method is adopted. In this method, the genome bits of the middle segments (between Positions 1 and 2) are inverted (i.e., if the genome bit is 1, then it is changed to 0 and vice versa), as presented in Fig 6(A).

For the second part (i.e., vector Y), the mutation operator is described, as illustrated in Fig 6(B). According to the mutation probability (*Pm*), minimal chromosomes are mutated from 3 to 1, 2, or 4.

#### Algorithm 2 –heuristic algorithm

The heuristic algorithm establishes the following main idea: first, the shortest path is searched, except the total service time restriction based on the virtual network. Then, the algorithm attempts to adjust the alternative transport tool from the last city pair to the first city pair to meet the total service time limitation. The detail of the heuristic algorithm is depicted as follows.

**Fig 7:**
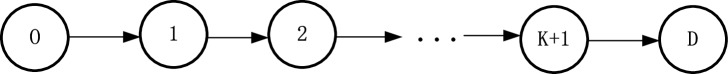
Original logistics service network.

**Fig 8:**
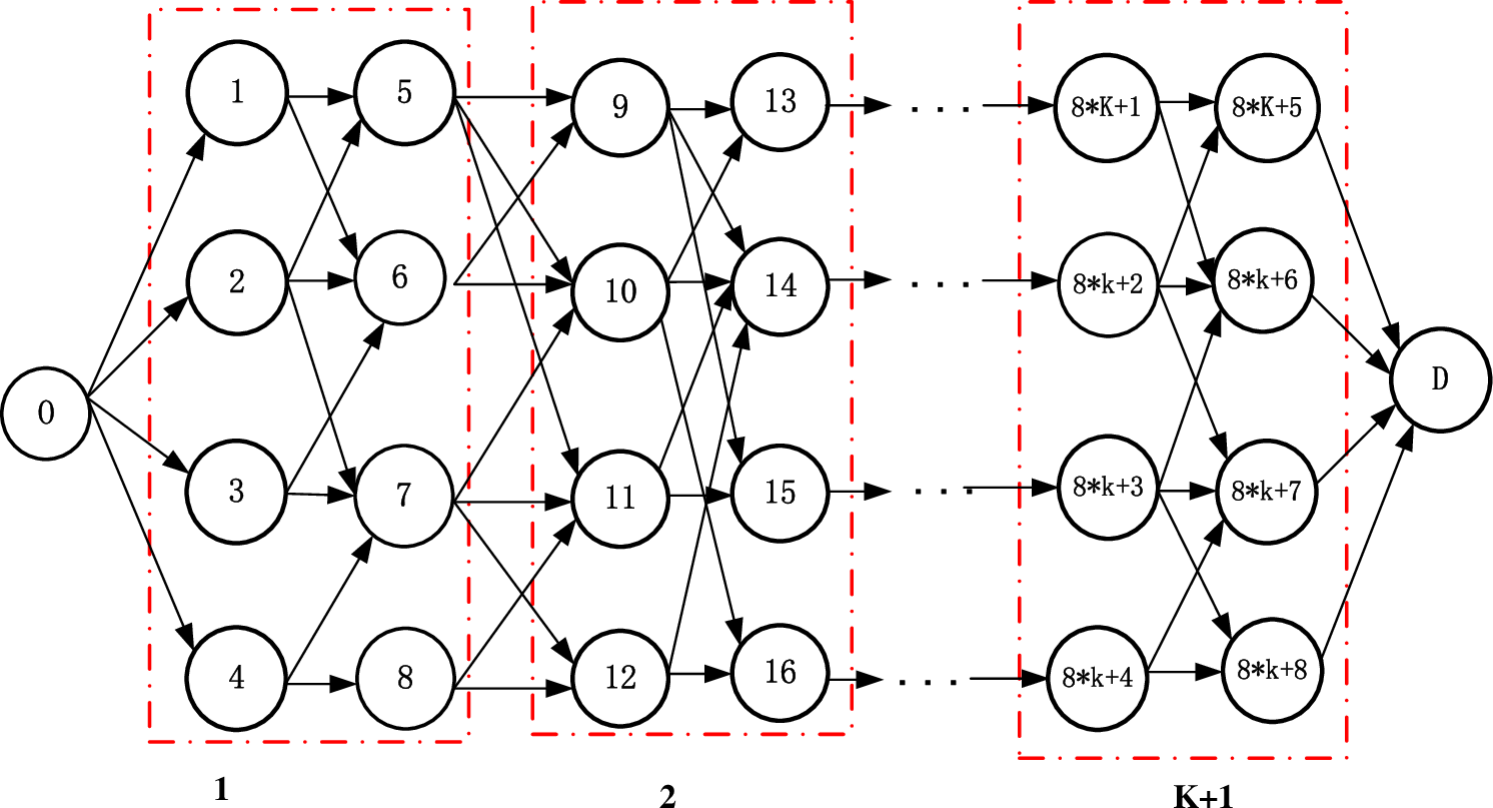
Virtual logistics service network.

Each link represents a transport mode for a given logistics service network from O to D via (K+1) city nodes (Fig 7), which can extend to a virtual logistics network (Fig 8).

The notation used in the algorithm is listed as follows:

Dist[*i*] is the shortest cost path from node *O* to node *i*.

Time[*i*] is the total time from node O to node *i*.

*W* is the actual total time of arrival at the destination city.

*t*_1_ and *t*_2_ represent the earliest and latest times of arrival at node D respectively.

Cost[*i*][*j*] is the cost between nodes *i* and *j*. If the transfer condition is unavailable, then cost[*i*][*j*] will be infinite.

*V*_*0*_ is the set of initial nodes; the virtual node set *V* = {*O*,1,2,3,⋯,(8*k+1),⋯,(8*k+8),*D*}; *S* is the set of labeled nodes; $\bar{S}$ is the set of unlabeled modes.

$S,\bar{S}\subset V$, and $S\cup \bar{S}=V$.

Path[*i*] = *k* indicates that the previous node of node *i* is node *k* along the shortest path from node *O* to node *i*. If no shortest path exists from node O to node *i*, then path[*i*] = infinite.

Label[*i*] = {0,1} implies whether the shortest path from node O to node *i* is found. If the label[*i*] is equal to l, then the shortest path from node *O* to node *i* is found; otherwise, 0.

Mode[*i*] represents the transport tool at the city pair *i*.

$\Delta T_{i}^{kl}$ is the time savings of changing the transport tool from tool *k* to tool *l* at city *i*.

$\Delta F_{i}^{kl}$ is the extra cost of changing the transport tool from tool *k* to tool *l* at city *i*, which involves the changes in transport costs because of the switch in the transport mode and investment cost to meet the feasibility of transferring the different transport tools at city *i*.

*Max*(*i*,*k*,l*) indicates that the transport tool *l* is the optimal tool among all available transport tools transferred from tool *k* at city *i*.

The outline of the algorithm is described as follows:

***Step 1*.** Initialization: *S* ← {O}; path[0] ← 0; label[0] = 1; $\bar{S}\leftarrow V-S$; dist[*i*] ← cost[0][*i*]; label[*i*] = 0; path[*i*] = ∞; ∀*i* ∈ *S*.

***Step 2*.** If label[*D*] = 1, then proceed to Step 6; otherwise, continue to Step 3.

***Step 3*.** Select min{dist[*j*]} and dist[*k*] = min{dist[*j*]}, where *i* ∈ *S*, $j\in \bar{S}$, and *q* ≤ *f*_*ij*_. Change $S,\bar{S}$ and $\mathrm{label}[k]:\bar{S}\leftarrow \bar{S}-\{k\},S\leftarrow S\cup \{k\},\mathrm{label}[k]=1$.

***Step 4*.** Dist[*j*]←min{dist[*i*][*j*],dist[i][*k*] + cost[*k*][*j*]}, $\mathrm{path}[j]\leftarrow k,\forall i\in S,j\in \bar{S}$; return to Step 2.

***Step 5*.** Output the shortest path list from node *O* to node *D*, and calculate the total transport time along the shortest path. Time *D* is the total time from node *O* to node *D*.

***Step 6*.** The transport tool at each city pair is obtained easily according to the shortest path found. Place the value into the array element mode[*i*] (*i* = 1,2,3……). Calculate the total transport time and cost: Total_time (*w*) = the total time used during the entire trip, including the transfer time; Total_cost (*Z*) = the total cost used during the entire trip, including transport, transfer, CO_2_ emission, and investment costs.

***Step 7***. If *t*_1_ ≤ *w* ≤ *t*_2_, then proceed to Step 9; otherwise, continue to Step 8.

***Step 8*.** *i* ← *n* + 1; *k* ← mode[*i*]; $\max\{i,k,l^{*}\}=\underset{l\in J}{\max}\{\frac{\Delta T_{i}^{kl}}{\Delta F_{i}^{kl}}\}$; w = w−$\Delta T_{i}^{kl}$; and Z = Z + $\Delta F_{i}^{kl}$, *i* ← *i* + 1; return to Step 2.

***Step 9*.** Output the final result, including the total time, total cost, and transport tool selected in each city pair and the investment decision at each transfer city.

In summary, the above algorithm is implemented in two stages. The first stage is to find the shortest path with no time restriction, and the second stage is to adjust time by changing transport modes to meet the time restriction using a heuristic method.

## Numerical example

### Input data and solution

**Fig 9:**
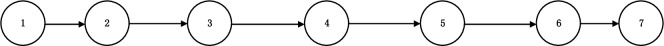
Multimodal logistics service network.

In the following subsection, a numerical example is used to illustrate the applications of the proposed model and solution algorithm. An example of the multimodal logistics service network is depicted in Fig 9. Four transport modes, namely, airway, highway, railway, and waterway, are available between each city pair in this example. The total weight of the logistics demand *Q* is assumed to be 20 tons. The time window of arrival at the end destination is set to [40, 50]. The carbon taxes are assumed to be $0.12/kg. Other parameters are displayed in Tables 1–5. In the following analysis, unless specifically stated otherwise, these input data are considered as the base case.

**Fig 10:**
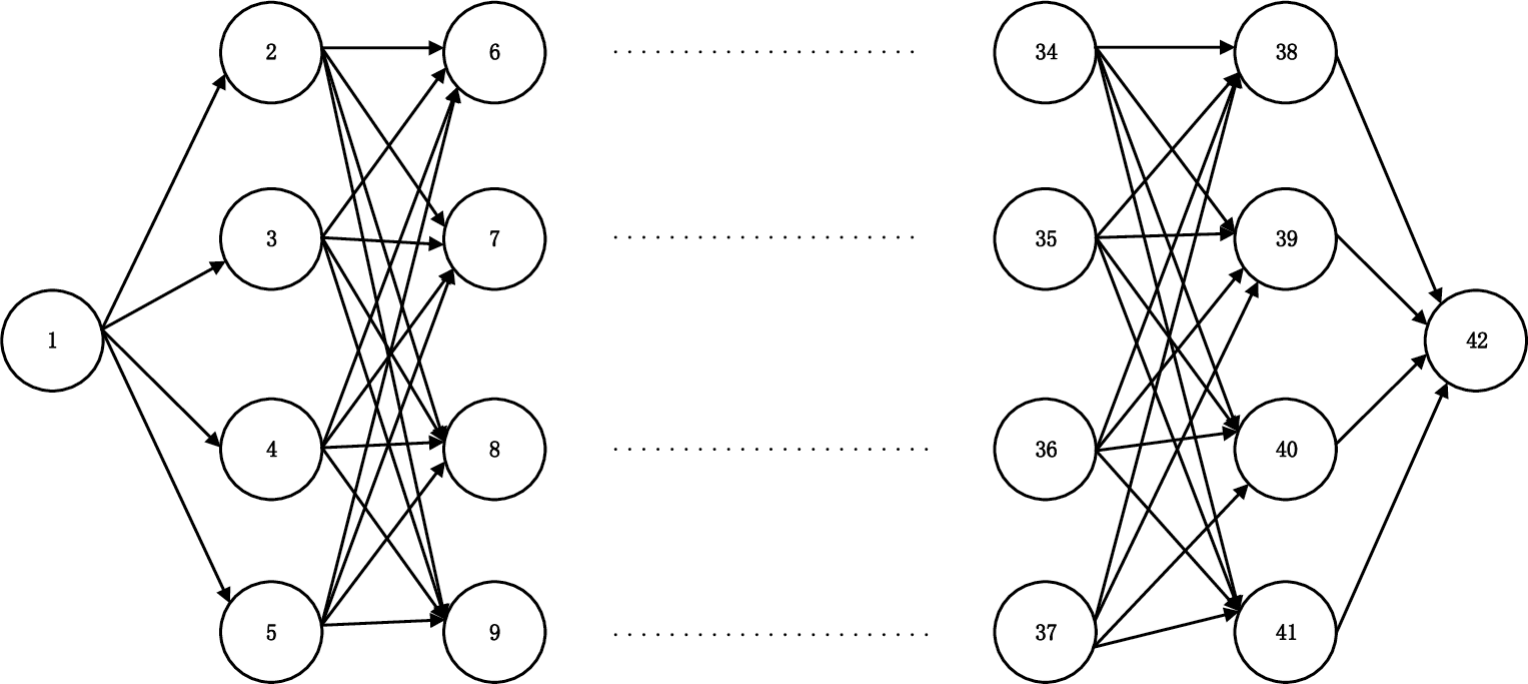
Multimodal logistics service network with added virtual nodes and links.

In Fig 10, several virtual nodes and links are added in the network to express the different transport modes between each node pair.

**Table 1:** Distance among city pairs (km).

City pair	(1, 2)	(2, 3)	(3, 4)	(4, 5)	(5, 6)	(6, 7)
Distance	500	1200	1300	1500	900	800

**Table 2:** Transport parameters of different transport modes.

Transport mode	Unit transport cost ($/t.km)	Unit carbon emission factor (kg/t.km)	Transport speed (km/h)
Airway	1.20	0.861	800
Highway	0.20	0.283	80
Railway	0.09	0.022	60
Waterway	0.06	0.016	30

(Source: Data adapted from references [31,37])

**Table 3:** Transfer cost ($/t) and time (h) among different transport modes.

	Airway	Highway	Railway	Waterway
Airway	0.0/0.0	1.2/1.5	1.3/2.0	1.5/3.0
Highway	1.2/1.5	0.0/0.0	0.8/1.0	1.0/1.2
Railway	1.3/2.0	0.8/1.0	0.0/0.0	0.6/1.8
Waterway	1.5/3.0	1.0/1.2	0.6/1.8	0.0/0.0

(Source: Data adapted from references [6,31])

**Table 4:** Transfer carbon emissions (kg/t) among different transport modes.

	Airway	Highway	Railway	Waterway
Airway	0.00	5.36	6.25	7.62
Highway	5.36	0.00	3.24	4.28
Railway	6.25	3.24	0.00	4.23
Waterway	7.62	4.28	4.23	0.00

**Table 5:** Transport capacity of different transport modes among city pairs (tons).

City pair	(1,2)	(2,3)	(3,4)	(4,5)	(5,6)	(6,7)
Airway	30	0	0	30	30	0
Highway	120	100	20	150	120	100
Railway	30	30	50	30	30	30
Waterway	200	0	300	0	200	300

The proposed solution algorithm is coded in Microsoft Visual C++ 6, which runs on a Lenovo ThinkPad T450 laptop with Intel Core i7 CPU and 8G RAM. The number of generations (i.e., stopping criterion) is set to 150. The optimal GA parameter crossover (Pc) and mutation rates (Pm) obtained are 0.7 and 0.1, respectively, after 20 runs. The iterative process of the GA for the reference case consumes approximately 1.96 s of CPU time. The final transport mode is 3-1-1-1-1-4. The total cost is $131732, and the CO_2_ emission is 85131.4 kg.

### Analysis and discussion

First, CO_2_ emission cost shows the effects on logistics service network design and their corresponding total costs and CO_2_ emissions. In Table 6, the optimal transport mode combinations and investment schemes differ from those without CO_2_ taxes in terms of CO_2_ emission cost. For example, the optimal transport mode combinations with CO_2_ emission tax charging are 2-1-1-1-1-3, which differ from those without CO_2_ taxes (i.e., the final transport combinations are 2-1-1-1-1-2) in terms of the service time window of [20, 30]. Moreover, the total costs are higher with CO_2_ emission charging than without CO_2_ emission charging, but the total CO_2_ emissions become less under the same service time windows. This finding indicates that transport mode combinations result in the low carbon combinations when we consider the carbon tax charging scheme.

**Table 6:** Different kinds of cost under different time windows with or without carbon tax charging.

Time window	CO_2_ emission tax charging	Transport mode choice	Investment selection	Total cost ($)	CO_2_ emissions (kg)
**[20, 30]**	NO	2-1-1-1-1-2	2,6	124848	91950
	YES	2-1-1-1-1-3	2,6	133625	87792
**[30, 40]**	NO	4-1-1-1-1-2	2,6	123454	89326
	YES	3-1-1-1-1-3	2,6	132216	85200
**[40, 50]**	NO	2-1-1-1-1-4	2,6	122614	87724
	YES	3-1-1-1-1-4	2,6	131732	85131
**[50, 60]**	NO	2-1-1-1-2-4	2,5,6	104628	77360
	YES	4-1-1-1-3-3	2,5	110124	70065
**[60, 70]**	NO	4-1-1-1-2-4	2,5,6	104234	74735
	YES	2-3-1-1-1-4	2,3,6	105112	67670
**[70, 80]**	NO	2-1-1-1-4-4	2,5	102094	72514
	YES	4-3-1-1-1-4	2,3,6	103390	65020

Next, we examine the effects of different service time windows on the optimal logistics service network design. In Table 7, the total costs and CO_2_ emissions decrease with the increase in the value of time service window interval. Specifically, the total cost and CO_2_ emissions decrease from $133625 and 87792 kg, respectively, under the time windows [20, 30] to $103390 and 65020 kg, correspondingly, under the time windows [70, 80].

**Table 7:** The optimal solutions of different service time windows.

Time window	Transport mode	investment selection	Total cost($)	CO_2_ emission (kg)	Investment cost ($)
**[20, 30]**	2-1-1-1-1-3	2,6	133625	87792	2000
**[30, 40]**	3-1-1-1-1-3	2,6	132216	85200	2000
**[40, 50]**	3-1-1-1-1-4	2,6	131732	85131	2000
**[50, 60]**	4-1-1-1-3-3	2,5	110124	70065	2000
**[60, 70]**	2-3-1-1-1-4	2,3,6	105112	67670	3000
**[70, 80]**	4-3-1-1-1-4	2,3,6	103390	65020	3000

**Fig 11:**
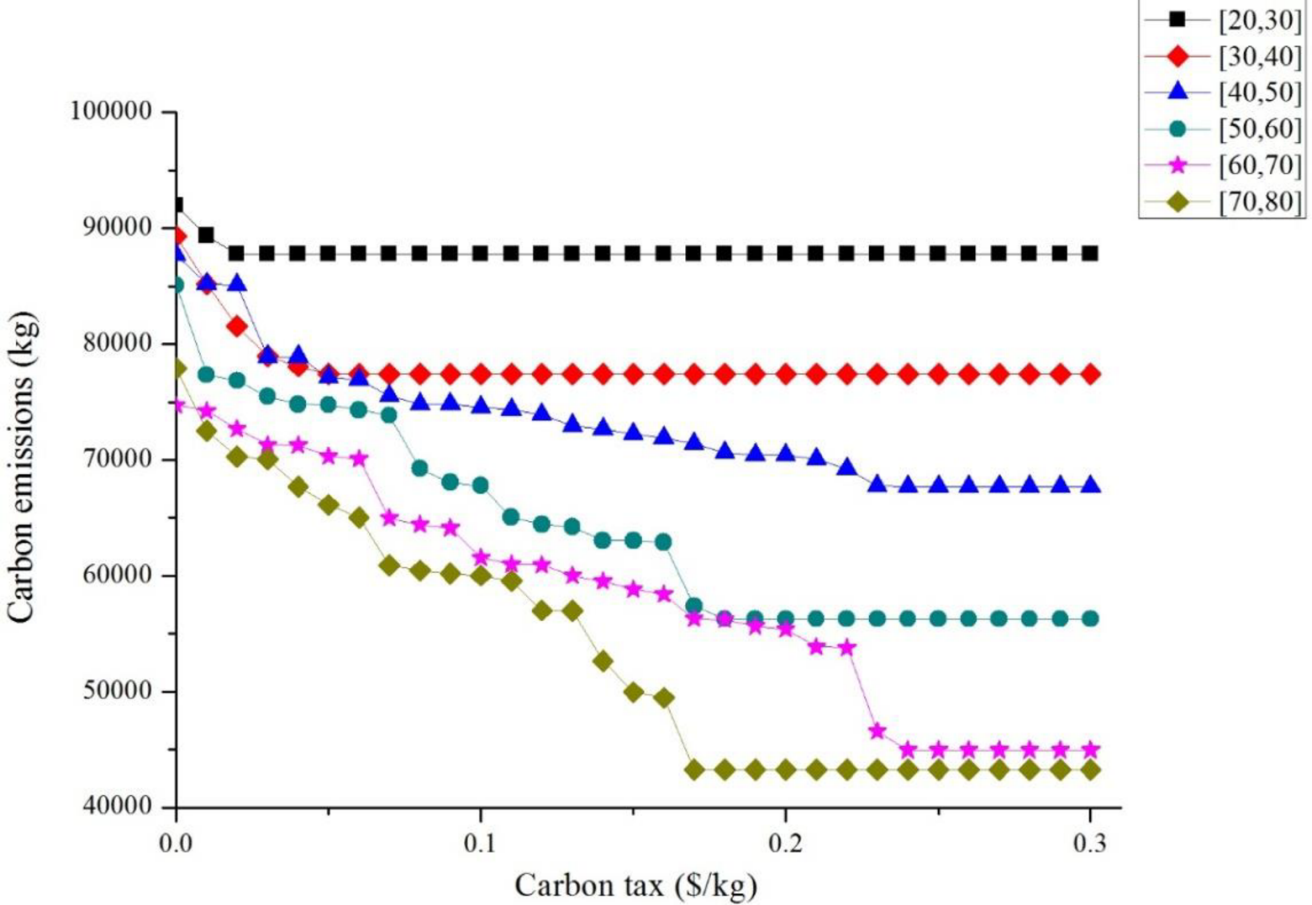
Carbon emissions under different carbon taxes of different time windows.

Furthermore, we address the effects of the different values of CO_2_ emission taxes charging. The change curve of the total carbon emissions under different carbon taxes is displayed in Fig 11. For a given logistics service time window, the total CO_2_ emission decreases with the increase in the value of CO_2_ emission tax, which reaches a minimal fixed value. This finding denotes that the saving of the total CO_2_ emissions remains unchanged with the increase in the CO_2_ emission tax charging. For example, the largest saving of CO_2_ emission is approximately 22.86% under the time window [40, 50].

**Fig 12:**
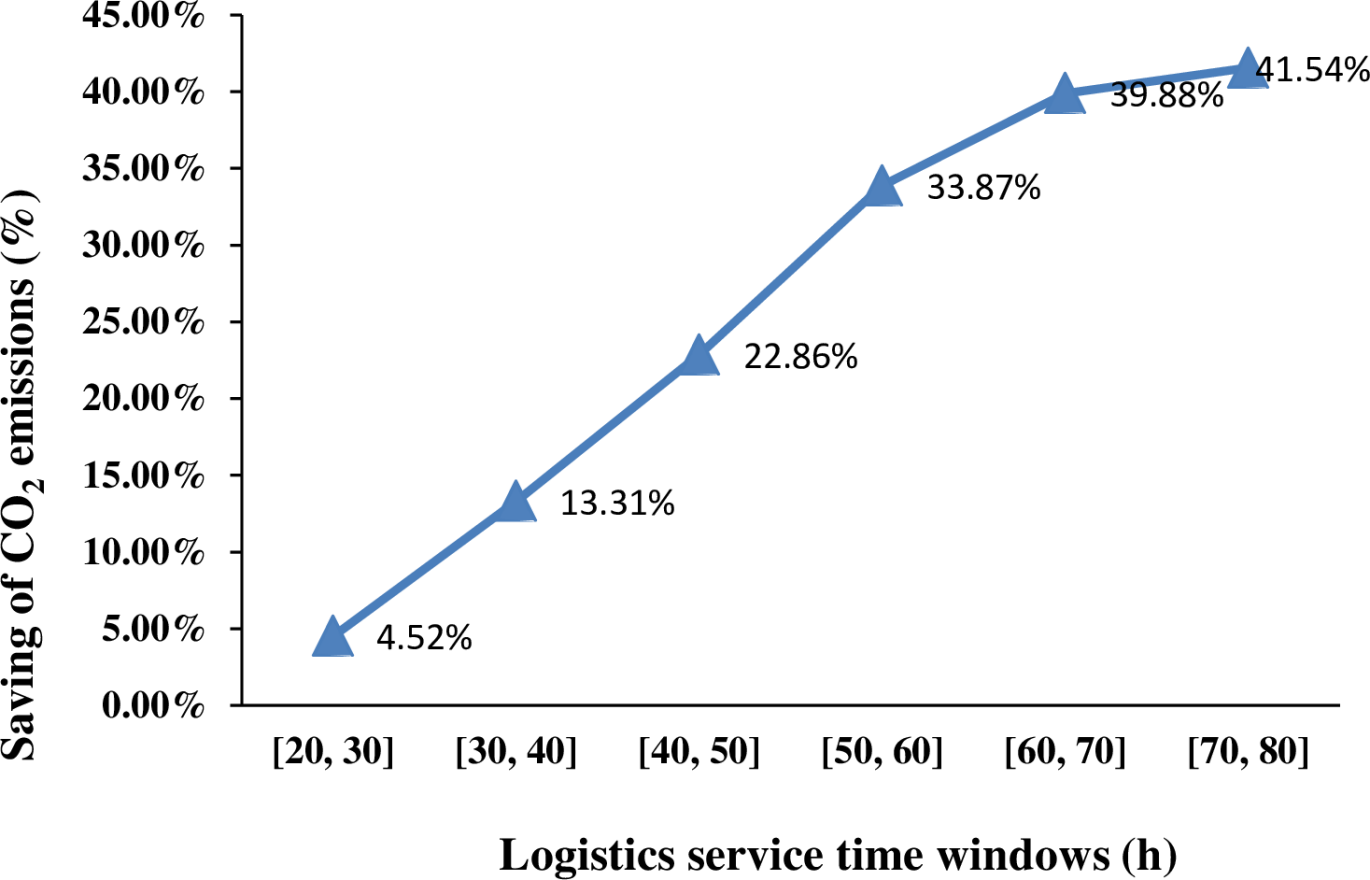
Saving of the total CO_2_ emissions under different time windows.

Finally, we compared the saving of the total CO_2_ emissions under different time windows. In Fig 12, the saving percentage of carbon emissions is approximately 4.52% when the time window is set to [20, 30]. The saving percentage of carbon emissions changed to 13.31% when the time window is changed to [30, 40]. Furthermore, the corresponding saving percentages changed to 39.88% and 41.54% when the time windows are changed to [60, 70] and [70, 80], respectively. In Fig 12, the saving percentage of carbon emissions increased gradually with the increase in the value of service time windows. This result indicates that the transport mode combinations can be switched to an improved green combination with the increase in the value of logistics service time windows.

### Algorithm comparisons

We present the results of our computational experiments to test the performance of the proposed GA and the heuristic algorithm. We generate six instances test data to validate the proposed algorithm considering the absence of publicly available instances for our research problem. The first instance input parameters are shown as Tables 1–5. And the detail of input parameters of other five test instances is shown in the Appendixes A-E in S1 File.

Each algorithm was executed 20 times, and the best solution was selected as the optimal logistics service network design results for each approach. The comparisons of the performance of the algorithm are presented in Table 8.

**Table 8:** Comparison of solutions between the GA, CPLEX 12.6, and heuristic algorithm.

Instances No.	|M|×|N|	CPLEX 12.6		Heuristic algorithm		GA	
		CPU (s)	Obj. ($)	CPU (s)	Obj. ($)	CPU (s)	Obj. ($)
1	6×4	3.65	131732.28	1.50	131732.28	1.96	131732.28
2	10×4	23.21	153732.70	3.25	153732.70	8.98	153732.70
3	15×4	581.12	256968.70	45.23	258978.20	95.32	256968.70
4	20×4	2018.56	345616.20	125.12	346328.80	587.23	345977.30
5	25×4	-	N/A	368.32	477983.56	1983.65	476136.45
6	30×4	-	N/A	783.17	568108.32	3208.17	562652.17

(Note: |M| and |N| represent the number of city pairs and transport modes, respectively)

Table 8 displays the following findings. The GA can obtain better optimization solutions than the heuristic algorithm, whereas the search for the optimal solutions requires more CPU time through the GA than through the heuristic algorithm. With increase of the size of the multimodal logistics service network design problem, the CPLEX solver cannot find the optimal solution because the computational complexity grows exponentially for the exact method in the large-scale multimodal logistics service network design with many transfer nodes. However, the computational times required by GA are acceptable. Thus, the GA algorithm is effective and efficient for extensive multimodal logistics service network design problems.

## Conclusions

In this work, we proposed a new optimal model of a multimodal logistics service network design considering transport time, transfer time, and external CO_2_ emission costs. Based on the characteristics of the optimization model, two related algorithms, namely, GA and heuristic algorithm are provided to solve the problem. The optimal model and corresponding algorithms were evaluated through a numerical example on the multimodal logistics service network design. The comparisons of the performance of the two algorithms are also analyzed. The results of numerical experiments indicate that the GA is more effective than the heuristic algorithm. Meanwhile, the following new insights and important findings are obtained from the numerical experiments:

(1) Different service time windows of customers significantly affect the logistics service network design, such as transport and transfer mode selections. A difficult time window limitation must specifically use a rapidly combined transportation service to decrease the transfer between the different transport modes in various cities with different transfer times and costs, which significantly influence the best route selection in the multimodal logistics service network.

(2) The transfer among different transport modes in different transfer nodes results in different transfer times and costs, which significantly influence the optimal selection of transfer nodes and transport modes in the multimodal logistics service network.

(3) The introduced CO_2_ emission taxes change the structure of the logistics service network, which aids in encouraging logistics users to switch to enhanced green logistics service paths using the corresponding combined transport modes.

(4) The transport mode combinations can be switched to an enhanced green combination with the increase in the value of logistics service time windows to obtain additional savings of CO_2_ emissions.

Limitations for further research were also identified. First, case studies on large and realistic logistics networks are necessary to further justify the findings of the present work and the performance of the proposed model, although the numerical results presented in this paper contain possible logical explanations. Second, the model can be extended as a robust model for addressing the multimodal logistics network design with uncertainty in the demand sides and the multi-period logistics network design and logistics service pricing problems.

## Supporting information

S1 File Details of input parameters of test instances. (S1-Appendixes.docx).
(DOCX)

## References

[1] Liotta G, Stecca G, Kaihara T (2015) Optimisation of freight flows and sourcing in sustainable production and transportation networks. International Journal of Production Economics 164: 351–365.

[2] SteadieSeifi M, Dellaert NP, Nuijten W, Van Woensel T, Raoufi R (2014) Multimodal freight transportation planning: A literature review. European Journal of Operational Research 233: 1–15.

[3] McKinnon AC, Cullinane S., Browne M., & Whiteing A. (2012) Green logistics: improving the environmental sustainability of logistics: Kogan Page Ltd.

[4] Piecyk MI, McKinnon AC (2010) Forecasting the carbon footprint of road freight transport in 2020. International Journal of Production Economics 128: 31–42.

[5] Dekker R, Bloemhof J, Mallidis I (2012) Operations Research for green logistics—An overview of aspects, issues, contributions and challenges. European Journal of Operational Research 219: 671–679.

[6] Zhang D, Zhan Q, Chen Y, Li S (2016) Joint optimization of logistics infrastructure investments and subsidies in a regional logistics network with CO2 emission reduction targets. Transportation Research Part D: Transport and Environment.

[7] Harris I, Mumford CL, Naim MM (2014) A hybrid multi-objective approach to capacitated facility location with flexible store allocation for green logistics modeling. Transportation Research Part E: Logistics and Transportation Review 66: 1–22.

[8] Ehmke JF, Campbell AM, Thomas BW (2016) Data-driven approaches for emissions-minimized paths in urban areas. Computers & Operations Research 67: 34–47.

[9] Fukasawa R, He Q, Song Y (2016) A disjunctive convex programming approach to the pollution-routing problem. Transportation Research Part B-Methodological 94: 61–79.

[10] Aronsson H, Brodin MH (2006) The environmental impact of changing logistics structures. International Journal of Logistics Management 17: 394–415.

[11] Elhedhli S, Merrick R (2012) Green supply chain network design to reduce carbon emissions. Transportation Research Part D-Transport and Environment 17: 370–379.

[12] Huang Y, Chen C-W, Fan Y (2010) Multistage optimization of the supply chains of biofuels. Transportation Research Part E-Logistics and Transportation Review 46: 820–830.

[13] Wang F, Lai X, Shi N (2011) A multi-objective optimization for green supply chain network design. Decision Support Systems 51: 262–269.

[14] Jindal A, Sangwan KS (2016) Multi-objective fuzzy mathematical modelling of closed-loop supply chain considering economical and environmental factors. Annals of Operations Research: 1–26.

[15] Santoso T, Ahmed S, Goetschalckx M, Shapiro A (2005) A stochastic programming approach for supply chain network design under uncertainty. European Journal of Operational Research 167: 96–115.

[16] Elhedhli S, Merrick R (2012) Green supply chain network design to reduce carbon emissions. Transportation Research Part D: Transport and Environment 17: 370–379.

[17] Kim D, Barnhart C (1997) Multimodal express shipment service design: Models and algorithms. Computers & Industrial Engineering 33: 685–688.

[18] Asgari N, Farahani RZ, Goh M (2013) Network design approach for hub ports-shipping companies competition and cooperation. Transportation Research Part a-Policy and Practice 48: 1–18.

[19] Ishfaq R, Sox CR (2011) Hub location-allocation in intermodal logistic networks. European Journal of Operational Research 210: 213–230.

[20] Chang T-S (2008) Best routes selection in international intermodal networks. Computers & Operations Research 35: 2877–2891.

[21] Tyan JC, Wang FK, Du TC (2003) An evaluation of freight consolidation policies in global third party logistics. Omega-International Journal of Management Science 31: 55–62.

[22] Wang Y, Ma XL, Xu MZ, Liu Y, Wang YH (2015) Two-echelon logistics distribution region partitioning problem based on a hybrid particle swarm optimization-genetic algorithm. Expert Systems with Applications 42: 5019–5031.

[23] Wang Y, Ma XL, Lao YT, Wang YH (2014) A fuzzy-based customer clustering approach with hierarchical structure for logistics network optimization. Expert Systems with Applications 41: 521–534.

[24] Wang Y, Ma XL, Liu MW, Gong K, Liu Y, et al (2017) Cooperation and profit allocation in two-echelon logistics joint distribution network optimization. Applied Soft Computing 56: 143–157.

[25] Haghani A, Oh SC (1996) Formulation and solution of a multi-commodity, multi-modal network flow model for disaster relief operations. Transportation Research Part A Policy & Practice 30: 231–250.

[26] Mohammadi M, Jula P, Tavakkoli-Moghaddam R (2017) Design of a reliable multi-modal multi-commodity model for hazardous materials transportation under uncertainty. European Journal of Operational Research 257: 792–809.

[27] Jansen B, Swinkels PCJ, Teeuwen GJA, de Fluiter BV, Fleuren HA (2004) Operational planning of a large-scale multi-modal transportation system. European Journal of Operational Research 156: 41–53.

[28] Bock S (2010) Real-time control of freight forwarder transportation networks by integrating multimodal transport chains. European Journal of Operational Research 200: 733–746.

[29] Alumur SA, Kara BY, Karasan OE (2012) Multimodal hub location and hub network design. Omega-International Journal of Management Science 40: 927–939.

[30] Moccia L, Cordeau J-F, Laporte G, Ropke S, Valentini MP (2011) Modeling and Solving a Multimodal Transportation Problem with Flexible-time and Scheduled Services. Networks 57: 53–68.

[31] Zhang D, Eglese R, Li S (2015) Optimal location and size of logistics parks in a regional logistics network with economies of scale and CO2 emission taxes. doi: 10.3846/16484142.2015.1004644 Transport.

[32] Harris I, Naim M, Palmer A, Potter A, Mumford C (2010) Assessing the impact of cost optimization based on infrastructure modelling on CO 2 emissions. International Journal of Production Economics 131: 313–321.

[33] Yang J, Guo J, Ma S (2016) Low-carbon city logistics distribution network design with resource deployment. Journal of Cleaner Production 119: 223–228.

[34] Wang Y, Zhu X, Lu T (2013) Eco-Efficient Based Logistics Network Design in Hybrid Manufacturing/Remanufacturing System Under Low-Carbon Restriction. Journal of Industrial Engineering & Management 6: 79–88.

[35] He Q, Luo W (2014) Low-carbon Closed-loop Logistics Network Design based on Interval Number Multi-attribute Decision and Queuing Theory. International Journal of Hybrid Information Technology 7: 235–248.

[36] Shaw K, Irfan M, Shankar R, Yadav SS (2016) Low carbon chance constrained supply chain network design problem: a Benders decomposition based approach. Computers & Industrial Engineering 98: 483–497.

[37] Qu Y, Bektas T, Bennell J (2016) Sustainability SI: Multimode Multicommodity Network Design Model for Intermodal Freight Transportation with Transfer and Emission Costs. Networks & Spatial Economics 16: 303–329.

[38] Paraskevopoulos DC, Guerel S, Bektas T (2016) The congested multicommodity network design problem. Transportation Research Part E-Logistics and Transportation Review 85: 166–187.

[39] Ganesh K, Punniyamoorthy M (2005) Optimization of continuous-time production planning using hybrid genetic algorithms-simulated annealing. International Journal of Advanced Manufacturing Technology 26: 148–154.

[40] Lopes RB, Ferreira C, Santos BS (2016) A simple and effective evolutionary algorithm for the capacitated location–routing problem. Computers & Operations Research 70: 155–162.

[41] Wang Z, Guo J, Zheng M, Wang Y (2015) Uncertain multiobjective traveling salesman problem. European Journal of Operational Research 241: 478–489.

[42] Zhang S, Lee CKM, Choy KL, Ho W, Ip WH (2014) Design and development of a hybrid artificial bee colony algorithm for the environmental vehicle routing problem. Transportation Research Part D: Transport and Environment 31: 85–99.

