# Supplementary material for: A multimodal logistics service network design with time windows and environmental concerns
Source: PLoS One. 2017 Sep 21;12(9):e0185001. doi: 10.1371/journal.pone.0185001 (PMC5608303; doi:10.1371/journal.pone.0185001)
Supplement: S1 File — (S1-Appendixes.docx). (DOCX) [file pone.0185001.s001.docx]

**Appendixes**

**Details of input parameters of test instance are shown as Appendixes A-E.**

# Appendix-A Detail of input parameters of instance-1 (n=10, m=4)

Table A1. Distance among city pairs (km)

| City pair | 1 | 2 | 3 | 4 | 5 | 6 | 7 | 8 | 9 | 10 |
| --- | --- | --- | --- | --- | --- | --- | --- | --- | --- | --- |
| Distance | 1116 | 1161 | 1441 | 1001 | 1436 | 813 | 970 | 1131 | 935 | 881 |

Table A2. Transport capacity of different transport modes among city pairs (tons)

| City pair | 1 | 2 | 3 | 4 | 5 | 6 | 7 | 8 | 9 | 10 |
| --- | --- | --- | --- | --- | --- | --- | --- | --- | --- | --- |
| Airway | 0 | 20 | 0 | 0 | 20 | 0 | 0 | 20 | 20 | 0 |
| Highway | 50 | 50 | 50 | 50 | 50 | 50 | 50 | 50 | 50 | 50 |
| Railway | 100 | 100 | 100 | 100 | 100 | 100 | 100 | 100 | 100 | 100 |
| Waterway | 0 | 200 | 0 | 200 | 0 | 0 | 0 | 200 | 200 | 200 |

The time window of arrival at the end destination is set to [90, 100].

The other input parameters are same as Tables 2-4*.*

# Appendix-B Detail of input parameters of instance-2 (n=15, m=4)

Table B1. Distance among city pairs (km)

| City pair | Distance |
| --- | --- |
| 1 | 1465 |
| 2 | 1124 |
| 3 | 809 |
| 4 | 1105 |
| 5 | 1219 |
| 6 | 1330 |
| 7 | 1246 |
| 8 | 1379 |
| 9 | 998 |
| 10 | 1401 |
| 11 | 934 |
| 12 | 1062 |
| 13 | 818 |
| 14 | 1263 |
| 15 | 1194 |

Table B2. Transport capacity of different transport modes among city pairs (tons)

| City pair | Airway | Highway | Railway | Waterway |
| --- | --- | --- | --- | --- |
| 1 | 0 | 50 | 100 | 0 |
| 2 | 20 | 50 | 100 | 200 |
| 3 | 0 | 50 | 100 | 0 |
| 4 | 0 | 50 | 100 | 200 |
| 5 | 20 | 50 | 100 | 0 |
| 6 | 0 | 50 | 100 | 0 |
| 7 | 0 | 50 | 100 | 0 |
| 8 | 20 | 50 | 100 | 200 |
| 9 | 20 | 50 | 100 | 200 |
| 10 | 0 | 50 | 100 | 200 |
| 11 | 20 | 50 | 100 | 0 |
| 12 | 0 | 50 | 100 | 0 |
| 13 | 20 | 50 | 100 | 200 |
| 14 | 20 | 50 | 100 | 0 |
| 15 | 0 | 50 | 100 | 200 |

The time window of arrival at the end destination is set to [140, 150].

The other input parameters are same as Tables 2-4.

# Appendix-C Detail of input parameters of instance-3 (n=20, m=4)

Table C1. Distance among city pairs (km)

| City pair | Distance | City pair | Distance |
| --- | --- | --- | --- |
| 1 | 1087 | 11 | 1288 |
| 2 | 1015 | 12 | 1029 |
| 3 | 1077 | 13 | 1150 |
| 4 | 1463 | 14 | 1341 |
| 5 | 907 | 15 | 1305 |
| 6 | 1470 | 16 | 1400 |
| 7 | 892 | 17 | 1430 |
| 8 | 823 | 18 | 847 |
| 9 | 1114 | 19 | 1270 |
| 10 | 818 | 20 | 858 |

**Table C2.** Transport capacity of different transport modes among city pairs (tons)

| City pair | Airway | Highway | Railway | Waterway |
| --- | --- | --- | --- | --- |
| 1 | 0 | 50 | 100 | 0 |
| 2 | 20 | 50 | 100 | 200 |
| 3 | 0 | 50 | 100 | 0 |
| 4 | 0 | 50 | 100 | 200 |
| 5 | 20 | 50 | 100 | 0 |
| 6 | 0 | 50 | 100 | 0 |
| 7 | 0 | 50 | 100 | 0 |
| 8 | 20 | 50 | 100 | 200 |
| 9 | 20 | 50 | 100 | 200 |
| 10 | 0 | 50 | 100 | 200 |
| 11 | 20 | 50 | 100 | 0 |
| 12 | 0 | 50 | 100 | 0 |
| 13 | 20 | 50 | 100 | 200 |
| 14 | 20 | 50 | 100 | 0 |
| 15 | 0 | 50 | 100 | 200 |
| 16 | 0 | 50 | 100 | 0 |
| 17 | 20 | 50 | 100 | 0 |
| 18 | 20 | 50 | 100 | 200 |
| 19 | 0 | 50 | 100 | 200 |
| 20 | 20 | 50 | 100 | 200 |

The time window of arrival at the end destination is set to [180, 190].

The other input parameters are same as Tables 2-4.

# Appendix-D Detail of input parameters of instance-4 (n=25, m=4)

Table D1. Distance among city pairs (km)

| City pair | Distance | City pair | Distance |
| --- | --- | --- | --- |
| 1 | 1261 | 14 | 1373 |
| 2 | 911 | 15 | 1140 |
| 3 | 1178 | 16 | 1171 |
| 4 | 1186 | 17 | 1459 |
| 5 | 995 | 18 | 1289 |
| 6 | 839 | 19 | 1384 |
| 7 | 1009 | 20 | 1197 |
| 8 | 913 | 21 | 1152 |
| 9 | 1231 | 22 | 1396 |
| 10 | 1161 | 23 | 929 |
| 11 | 1053 | 24 | 1311 |
| 12 | 829 | 25 | 1294 |
| 13 | 1404 |  |  |

**Table D2.** Transport capacity of different transport modes among city pairs (tons)

| City pair | Airway | Highway | Railway | Waterway |
| --- | --- | --- | --- | --- |
| 1 | 0 | 50 | 100 | 0 |
| 2 | 20 | 50 | 100 | 200 |
| 3 | 0 | 50 | 100 | 0 |
| 4 | 0 | 50 | 100 | 200 |
| 5 | 20 | 50 | 100 | 0 |
| 6 | 0 | 50 | 100 | 0 |
| 7 | 0 | 50 | 100 | 0 |
| 8 | 20 | 50 | 100 | 200 |
| 9 | 20 | 50 | 100 | 200 |
| 10 | 0 | 50 | 100 | 200 |
| 11 | 20 | 50 | 100 | 0 |
| 12 | 0 | 50 | 100 | 0 |
| 13 | 20 | 50 | 100 | 200 |
| 14 | 20 | 50 | 100 | 0 |
| 15 | 0 | 50 | 100 | 200 |
| 16 | 0 | 50 | 100 | 0 |
| 17 | 20 | 50 | 100 | 0 |
| 18 | 20 | 50 | 100 | 200 |
| 19 | 0 | 50 | 100 | 200 |
| 20 | 20 | 50 | 100 | 200 |
| 21 | 0 | 50 | 100 | 200 |
| 22 | 0 | 50 | 100 | 0 |
| 23 | 20 | 50 | 100 | 0 |
| 24 | 20 | 50 | 100 | 200 |
| 25 | 20 | 50 | 100 | 200 |

The time window of arrival at the end destination is set to [210, 220].

The other input parameters are same as Tables 2-4.

# Appendix-E Detail of input parameters of instance-5 (n=30, m=4)

Table E1. Distance among city pairs (km)

| City pair | Distance | City pair | Distance |
| --- | --- | --- | --- |
| 1 | 1161 | 16 | 1318 |
| 2 | 1017 | 17 | 1202 |
| 3 | 981 | 18 | 962 |
| 4 | 1404 | 19 | 961 |
| 5 | 1269 | 20 | 1126 |
| 6 | 1452 | 21 | 1044 |
| 7 | 1415 | 22 | 1027 |
| 8 | 1407 | 23 | 1074 |
| 9 | 892 | 24 | 1425 |
| 10 | 1061 | 25 | 958 |
| 11 | 1124 | 26 | 1154 |
| 12 | 1125 | 27 | 872 |
| 13 | 1079 | 28 | 983 |
| 14 | 1239 | 29 | 918 |
| 15 | 969 | 30 | 1372 |

**Table E2.** Transport capacity of different transport modes among city pairs (tons)

| City pair | Airway | Highway | Railway | Waterway |
| --- | --- | --- | --- | --- |
| 1 | 0 | 50 | 100 | 0 |
| 2 | 20 | 50 | 100 | 200 |
| 3 | 0 | 50 | 100 | 0 |
| 4 | 0 | 50 | 100 | 200 |
| 5 | 20 | 50 | 100 | 0 |
| 6 | 0 | 50 | 100 | 0 |
| 7 | 0 | 50 | 100 | 0 |
| 8 | 20 | 50 | 100 | 200 |
| 9 | 20 | 50 | 100 | 200 |
| 10 | 0 | 50 | 100 | 200 |
| 11 | 20 | 50 | 100 | 0 |
| 12 | 0 | 50 | 100 | 0 |
| 13 | 20 | 50 | 100 | 200 |
| 14 | 20 | 50 | 100 | 0 |
| 15 | 0 | 50 | 100 | 200 |
| 16 | 0 | 50 | 100 | 0 |
| 17 | 20 | 50 | 100 | 0 |
| 18 | 20 | 50 | 100 | 200 |
| 19 | 0 | 50 | 100 | 200 |
| 20 | 20 | 50 | 100 | 200 |
| 21 | 0 | 50 | 100 | 200 |
| 22 | 0 | 50 | 100 | 0 |
| 23 | 20 | 50 | 100 | 0 |
| 24 | 20 | 50 | 100 | 200 |
| 25 | 20 | 50 | 100 | 200 |
| 26 | 0 | 50 | 100 | 0 |
| 27 | 0 | 50 | 100 | 200 |
| 28 | 20 | 50 | 100 | 200 |
| 29 | 20 | 50 | 100 | 200 |
| 30 | 20 | 50 | 100 | 0 |

The time window of arrival at the end destination is set to [240, 250].

The other input parameters are same as Tables 2-4.
